# The Role of Perceived Social Support in Assessing Posttraumatic Stress Disorder and Mental Health-Related Quality of Life in Veterans

**DOI:** 10.3390/healthcare8040396

**Published:** 2020-10-12

**Authors:** Melita Jukić, Ana Marija Lukinac, Ivan Požgain, Jasminka Talapko, Marko Jukić, Pavo Filaković

**Affiliations:** 1 Department of Psychiatry, County General Hospital Vukovar and Croatian Veterans’ Hospital, 32000 Vukovar, Croatia; mjuki17@gmail.com; 2 Faculty of Medicine, Josip Juraj Strossmayer University of Osijek, 31000 Osijek, Croatia; lukinac28@gmail.com; 3 Department of Rheumatology, Clinical Immunology and Allergology, University Hospital Center Osijek, 31000 Osijek, Croatia; 4 Psychiatric Clinic, University Hospital Center Osijek, 31000 Osijek, Croatia; pozgain.ivan@kbo.hr; 5 Faculty of Dental Medicine and Health, Josip Juraj Strossmayer University of Osijek, 31000 Osijek, Croatia; jtalapko@fdmz.hr (J.T.); pavo.filakovic@fdmz.hr (P.F.); 6 Faculty of Food Technology Osijek, Josip Juraj Strossmayer University of Osijek, 31000 Osijek, Croatia

**Keywords:** post-traumatic stress disorder, mental health-related quality of life, self-perceived social support, veterans, ex-prisoners of war

## Abstract

This study aims to establish the effect of self-perceived social support on the intensity of Post-Traumatic Stress Disorder (PTSD) symptoms and Mental Health-Related Quality of Life (MHRQoL) in veterans more than two decades after exposure to trauma in the Homeland War in Croatia, which took place from 1990 to 1995. The sample comprised 259 Croatian Homeland War veterans diagnosed with PTSD, with at least 6 months of combat experience. Among them, 90 subjects had also experienced imprisonment in enemy prison camps (at least 1 month of captivity). The subjects were evaluated using the questionnaire on self-perceived social support, sociodemographic questionnaire, PTSD self-report checklist (PCL-5) and Short Form (SF-36) Health Survey questionnaire. A general regression model analysis was performed to determine whether social support affected patients’ MHRQoL and intensity of the PTSD symptoms. The obtained results showed that veterans who had a more positive perception of social support after the events of the war had less intense PTSD symptoms and better MHRQoL. Furthermore, captivity and socioeconomic status were shown to be important predictors of PTSD and MHRQoL. The nonimprisoned veteran group was more likely to develop more severe PTSD symptoms and have poorer MHRQoL compared to the group of former prisoners of war (ex-POWs). This could be due to better post-war care and social support, which ex-POWs received after their release from captivity.

## 1. Introduction

The consequences of traumatic events experienced during wars are the subject of a great deal of research. Particular attention is focused on post-traumatic stress disorder (PTSD) and the factors that favor its development, or those that have a protective effect. According to the International Classification of Diseases (ICD-10), the main symptoms of PTSD are repeated reliving of the trauma, avoidance and detachment behavior, and hyperarousal with hypervigilance. In addition to the above, in the Diagnostic and Statistical Manual of Mental Disorders (DSM-5), there is also a group of symptoms, including cognitive and mood disturbances. Negative alterations in cognitions and mood can be expressed as the inability to remember some parts of the traumatic event, negative attitude and emotions, blaming oneself, reduced interest in significant activities, feelings of detachment from others, and a persistent lack of positive emotions [1,2].

The incidence of PTSD in war veterans varies depending on which veteran population it refers to and ranges widely. Epidemiological studies have mentioned a lifetime prevalence of PTSD in veterans with combat experience of about 30% [3]. In Croatia, there has been no systematic epidemiological research into the prevalence of PTSD in veterans, but research that included more than 3000 veterans of the Homeland War, which took place in the Republic of Croatia from 1991 to 1995, showed that 16.2% of veterans had PTSD, while 25% had partial PTSD [4]. Studies dealing with the consequences of imprisonment indicated the high prevalence of PTSD in ex-prisoners of war (ex-POWs) [5]. For instance, in ex-POWs from the Korean War, the prevalence was 88% [6]. Research has shown the persistence of PTSD, even many decades after exposure to war trauma. Research into ex-POWs from the World War II showed that the frequency of PTSD was about 50% at 40 years after imprisonment [7]. A study including Vietnam War veterans indicated more significant symptoms of PTSD 40 years after the war than 25 years earlier [8]. The series of factors that cause the development of PTSD can be divided into pre-traumatic (age, gender, earlier traumatization, educational and socioeconomic status, personality, earlier mental disorders, positive family history of mental disorders), peri-traumatic (the characteristics of the trauma, peri-traumatic emotions, peri-traumatic disassociation), and post-traumatic (social support and later life stressors) [9].

Many studies have dealt with the significance of social support in relation to the occurrence and intensity of PTSD and found that social support is one of the most powerful predictors of the intensity of symptoms of PTSD [10,11]. Social support is defined as the network of psychological and material resources available to the individual in facing stress, and there are various types of social support: Structural, functional, emotional, instrumental/material, and informational [12]. According to another study, the types of social support are: 1. Emotional support, which is aimed at strengthening self-respect in traumatized persons, along with the support and creation of a feeling of being appreciated and accepted by the environment; 2. Informational support, which helps in understanding the stressful event and facing it through giving advice, cognitive direction, and support in evaluation; 3. Support through social contacts, which encourages positive emotions and directing thoughts and activities away from difficulties; 4. Instrumental support, including financial and other material forms of support, providing services necessary to resolve the problems of traumatized people [13]. Perceived social support is perhaps the most important aspect of social support, and it relates to a person’s conviction that they will receive support when they need it. It is the recognized availability of people who make an individual feel cared for and supported. Perceived social support has a more important role in the psychological well-being and positive outcomes of some traumatic events in comparison to large social networks (of those who provide support), or the type of support offered [12]. Perceived support has a strong effect on all measures of health. People with high levels of self-perceived social support are usually more psychologically healthy. A number of studies have confirmed the effect of PTSD on the quality of life (QoL). Apart from mental disorders, the quality of life is also affected by the level of social support [14,15]. The World Health Organization (WHO) defines the QoL as an individual’s perception of their own position in a specific health, cultural, social, and environmental context. The Health-Related Quality of Life (HRQoL) is defined as “an integrative measure of physical and emotional well-being, level of independence, social relationships and their relationship to the salient features of their environment” [16].

This study aimed to establish the effect of self-perceived social support after exposure to war trauma on the intensity of symptoms of PTSD and Mental Health-Related Quality of Life (MHRQoL) more than two decades after the end of the Homeland War in Croatia. All subjects were diagnosed with PTSD. The study was part of wider research dealing with the mental and somatic health of ex-POWs from the Homeland War in the Republic of Croatia, which sought to identify potential differences among various health indicators between imprisoned and nonimprisoned veterans.

## 2. Materials and Methods

### 2.1. Subjects

The sample comprised 259 Croatian war veterans who were treated in 2017 at the Psychiatric Department of the County General Hospital Vukovar and Croatian Veterans’ Hospital with a diagnosis of PTSD, on the basis of the diagnostic criteria of the ICD-10 [1]. All subjects were male, had combat experience, and were active participants in the defense of the Republic of Croatia during the Homeland War (for at least 6 months from 1990 to 1995). Among them, 90 subjects also had experienced imprisonment in enemy prison camps (at least 1 month of captivity). All subjects gave informed consent to participate in the study, and patient anonymity was preserved at all stages of the study. When signing the informed consent, all subjects received a code that was further used in the questionnaires. The survey data with the special code was stored separately from the personal information that identified participants. In this way, it was ensured that the personal data were known only to their personal physician. A clinical interview was conducted with all the subjects at the psychiatry department by specialists in general psychiatry with subspecialty endorsement in social and biological psychiatry. The exclusion criteria from the study were: 1. Current psychotic and other mental disorders that, due to the severity and nature of the disorder, interfered with a person’s understanding and cooperation in the examination; 2. Physical illness or poor general condition that prevented a person from participating in research; 3. Failure to provide consent to participate in the study or any reason for withdrawal from the examination after having previously consented. A total of 284 patients were asked for consent in the study, of which 259 gave their consent, leading to a participation rate of 91%. The study was approved by the Ethics Committee of the Faculty of Medicine in Osijek (Code: 602-04/17-08/12) and by the Ethics Committee of the County General Hospital Vukovar and Croatian Veterans’ Hospital (Code: 510-05/17).

### 2.2. Questionnaires

A questionnaire on perceived social support was constructed for the needs of this research (Figure S1). It consisted of five questions relating to the social support of the: 1. Family, 2. Close friends, 3. Other important people in life, 4. Comrades or veteran organizations, and 5. Social community upon return from the war. Yes and no answers were possible for all the questions. Subjects were divided into three groups on the basis of how they evaluated their social support upon their return from the war or imprisonment. On the basis of their responses, the veterans were assigned to groups with low, moderate, or high self-perceived levels of social support. Those who did not have support in any of the categories offered, or in just one category, were designated as the group with low social support, those with perceived social support in two or three categories offered were designated as the group with moderate support, and those with four or all five categories were designated as the group with a high level of social support (Table 1).

A sociodemographic questionnaire was used containing questions on age, employment status, education, marital status, and self-assessment of socioeconomic status. This questionnaire also contained a question about imprisonment in enemy camps and its duration.

The PCL-5 (PTSD checklist) was used as a self-report measure whereby subjects assess themselves for individual symptoms of PTSD on the basis of the DSM-5 criteria [2,17]. The checklist contained 20 items related to specific symptoms in the past month. Items were rated on a five-point scale, and four clusters (subscales) were created. Items 1–5 was combined to form cluster B-Intrusion, items 6 and 7 formed C-Avoidance, items 8–14 D-Negative alterations in cognitions and mood, and items 15–20 E-Alterations in arousal and reactivity, respectively [2,18]. By summing the scores for each item, the total PCL-5 score was calculated. The total PCL-5 score ranged from 0 to 80, where higher scores indicated more severe symptoms [19].

The Short Form (SF-36) Health Survey questionnaire, containing 36 items (questions), was also used. Only the part of the questionnaire that relates to the mental component of health was used for the purposes of this study, that is, the psychological health profile, which includes four dimensions of mental health. The dimensions of the Mental component of health included: Vitality (VT), Social functioning (SF), Emotional role limitations (RE), and Mental health (MH). The original scores were adjusted by summing and rescaling the factor-weighted scores to fit the four dimensions of the Mental component of health with a range from 0 to 100, where a score of 100 indicated the best health condition. These four dimensions of health have also been distributed and summarized in the Mental component score (MCS) [20].

The internal consistency of the questionnaires was evaluated with Cronbach’s alpha coefficients. The Cronbach’s alpha for the questionnaire on perceived social support was 0.71 and was regarded as satisfactory. The Cronbach’s alpha coefficients for PCL-5 subscales were 0.90, 0.87, 0.85, and 0.86 for the B, C, D, and E subscales, respectively. The Cronbach’s alpha for the dimensions of the Mental component of the SF-36 questionnaire were 0.73, 0.76, 0.84, and 0.75 for VT, SF, RE, and MH, respectively.

### 2.3. Statistical Analyses

The descriptive statistics were calculated for sociodemographic data (frequencies and percentages) and for data on PCL-5 and SF-36 questionnaires (subscales means and standard deviations (SD)). Chi-square tests (*χ*^2^) for categorical variables and the Kruskal–Wallis H test (followed by multiple pairwise comparisons using Dunn’s procedure with Bonferroni correction) for quantitative measures were computed to compare groups with low, moderate, and high levels of perceived social support, with a significance level equal to 0.05.

A general regression model analysis was performed to determine whether social support affected patients’ QoL and intensity of the PTSD symptoms. First, univariate analysis was conducted to estimate the relationships between the indicators of PTSD and MHRQoL and the self-perceived social support as a predictor. Second, the regression model was expanded with the use of sociodemographic data to establish a possible confounding impact of these factors. A regression analysis was performed with the assumptions that relationship between responses and predictors was linear, errors were normally distributed, and observations were independent.

Statistical analysis was conducted using the TIBCO Statistica, v. 13.4 (TIBCO Software Inc., Palo Alto, CA, USA).

## 3. Results and Discussion

### 3.1. Sociodemographic Data and Data on Imprisonment

Earlier, in the first part of the research dealing with the mental and somatic health of ex-POWs from the Homeland War in the Republic of Croatia, we examined the intensity of PTSD symptoms in veterans treated for the diagnosis of PTSD, where we compared the intensity of symptoms and the level of the HRQoL in war veterans who were prisoners of war and those who did not have the experience of imprisonment. Overall, in contrast to our expectations, more than two decades after their exposure to war trauma, ex-POWs were in better condition than the nonimprisoned veterans regarding the PTSD and HRQoL indicators [19]. From clinical experience with veterans and the information obtained, it can be concluded that most of the ex-POWs, especially those from the Vukovar area, received some organized state assistance after leaving the detention camp (most of them were exchanged in 1992). Various psychosocial programs were organized for ex-POWs and their families. Most of them received psychological, medical, material, accommodation, and legal assistance. At the same time, the war was still going on in Croatia, and a large number of veterans who were not imprisoned were still at the battlefield (most of them until 1995). In line with the results, the question arose whether the social support for veterans affects the intensity of PTSD symptoms. Thus, in this study, we examined the possible influence of perceived social support on the expression of PTSD and MHRQoL.

A total of 259 subjects participated in this survey, of which 160, 70, and 29 subjects reported a low, moderate, and high level of perceived social support, respectively (Table 1). Data on the sociodemographic characteristics and imprisonment of participants are presented in Table 2. There were no statistically significant differences between the groups in terms of employment and marital status. The largest number of subjects were aged between 45 and 64 years, most of them had completed high school education, and most of the subjects were married. A significant difference (*χ*^2^ = 9.760; *p* = 0.008) was found between the groups in terms of imprisonment, primarily as a result of the higher percentage of nonimprisoned veterans with a perception of low social support compared to ex-POWs. This may be related to the fact that the ex-POWs actually had better support after returning from detention, especially by government institutions. The relevance of social support was pointed out by the results of a number of other studies [21,22]. The level of social support was shown to be a more significant predictor of HRQoL than sociodemographic or medical factors, and veterans with a high level of encouraging social support had better HRQoL. According to some studies, it has been found that, like the veterans who were exposed to traumatic combat experience without the experience of imprisonment, social support also proved to be extremely important for ex-POWs. In the process of recovery from the trauma of imprisonment, social support plays a major role [23]. As the most important period that determines a positive or negative outcome in terms of mental health, ex-POWs precisely identified the period of their return home after release from imprisonment [24]. In contrast, a study regarding prisoners from the Yom Kippur War described how they received partial social support after their return when they were welcomed sincerely but with extreme suspicion and distrust by the Israeli defense forces. This had extremely negative consequences and led to the intensification of the negative effects of imprisonment, destroying the feeling of belonging and connection in the prisoners, which had helped them face the situation and gave them strength during their imprisonment [25].

There were statistically highly significant differences between the groups in terms of their self-assessment of socioeconomic status (*χ*^2^ = 114.997; *p* < 0.001). In the group with a low level of perceived social support, the largest number assessed their socioeconomic status as average (*N* = 93), and the fewest assessed their socioeconomic status as good (*N* = 4). In the group with a moderate level of social support, again, the largest percentage assessed their socioeconomic status as average (*N* = 58), and an equal number of participants assessed it as good or bad (*N* = 6). In the group with a high assessment of social support, there were no participants who assessed their socioeconomic status as bad (*N* = 0), in comparison to the number of those with an average (*N* = 11) and good socioeconomic status (*N* = 18). These results showed that the perception of social support was closely related to the socioeconomic status of veterans. This association between perceived social support and socioeconomic status has been found in a number of other studies [26,27,28].

### 3.2. Social Support, PTSD, and MHRQoL

To date, research has shown that upon their return from the war, many veterans have difficulties becoming involved in civilian life, often experience complete social isolation, and feel that they are unable to share their traumatic experiences with others or experience a negative reaction in their environment [29]. War veterans are very sensitive to the way the environment treats them and how society reacts to their problems [30]. More social support upon the return from a war is linked with less intense symptoms of PTSD [31].

Table 3 shows a comparison of the groups with different perceptions of social support regarding the PCL-5 cluster scores and dimensions of the Mental component of health of the SF-36 questionnaire. According to the Kruskal–Wallis H test, there were significant differences between groups in all observed indicators of PTSD symptoms and MHRQoL. The group with a low perception of social support after the events of the war (including imprisonment) had significantly higher scores for PCL-5 clusters, indicating more severe PTSD symptoms. The results indicated social support as an important protective factor for the development of PTSD, and that veterans with lower social support had more symptoms of the disorder many years after exposure to combat experience.

These results are in line with many other studies. In research dealing with the relationship between social support and suicide risk in veterans of the Afghanistan War suffering from PTSD, where PTSD is a potentially moderating factor in this relationship, the results indicated that the risk of suicide was lower in those who were satisfied with their network of social relationships, but PTSD was a factor that could reduce this protective influence of social support [32]. A study including 363 veterans from the World War II confirmed the connection between a low level of perceived social support and high intensity of PTSD symptoms. Here, the perceived social support referred to the availability of persons who can meet the specific needs of veterans, including the need for love, counselling etc. [33]. In a retrospective study including veterans of the Vietnam War, a comparative analysis of social support over three periods of life showed that there was a decrease in social support over time for veterans suffering from PTSD (in contrast to those who did not suffer from PTSD), and that a higher level of social support were linked to a lower level of symptoms of traumatic stress in the post-war period [29].

PTSD may have a negative effect on the provision of social support to veterans [34]. In our study, the Avoidance (C) cluster had the highest score among the four criteria of PTSD in DSM-5 (mean values 2.4, 2.0, and 1.7 in the groups of low, moderate, and high perception of social support, respectively). This could be an indicator of the possible problems in attempts to provide some types of social support. Avoidance, as a symptom of PTSD, can lead to a refusal to seek help. Therefore, some veterans avoid treatment and rehabilitation programs in order to avoid other veterans and to not have to talk about their traumatic experiences. Similarly, research dealing with the connection between the severity of PTSD symptoms and social support in veterans of the Gulf War showed that PTSD had an erosive effect on the provision of social support [35].

PTSD has a significant negative effect on HRQoL and is associated with many health problems and poorer health perceptions in general [36,37]. The importance of assessment of HRQoL is shown by the fact that HRQoL is more significantly correlated with mortality than some objective health measurements [38]. In the same way, more intense symptoms of PTSD and the perception of a lower quality of life may lead to the perception of poorer accessibility of social support [39]. The results presented in Table 3 showed significant differences among groups in the results of the SF-36 questionnaire in the dimensions relating to mental health. Again, as for the PCL-5 questionnaire, the group with a low perception of social support had significantly lower scores for all four mental health dimensions, indicating poorer QoL of these subjects. The smallest but still significant difference was determined for the Vitality dimension (*H* = 13.532; *p* = 0.001), while the dimension of Social functioning was the factor in which differences related to perceived social support between groups were most pronounced (*H* = 51.988; *p* < 0.001). The highest scores for emotional role limitations and mental health dimensions were observed in the group with a high perception of social support, followed by the group with a moderate perception of social support. Overall, the highest score for the Social functioning dimension was in the group with a high perception of social support (61.2). Similar results have been obtained in a number of other studies that have dealt with the influence of perceived social support on various aspects of veterans’ health. Studies dealing with the effects of various types of social support on health, including support from public institutions and family support, have confirmed a significant correlation between these variables, as well as the influence of social support on psychopathology and social functioning [40,41,42]. The influence of social support is important for a feeling of well-being and its positive effect on health and reducing distress [43]. Also, perceived social support had a stronger effect on QoL than received social support, and it is possible that more mentally healthy people perceive social support more positively and have better social skills in seeking social support [15].

There were statistically highly significant differences between the groups in the Mental component scores (*H* = 55.005; *p* = 0.001). Cumulative values for Mental component scores were smallest in the group with low perception of social support (24.7), compared to groups with moderate and high perception of social support (35.0 and 45.2, respectively) (Table 3). In contrast, research into the relationship between social support and HRQoL in veterans suffering from PTSD and depression showed that greater social support would not ease the effect of PTSD and depression in terms of the Mental component score, but there was a clear positive correlation between social support and the Mental component score, that is, the stronger the social support, the higher the Mental component score [44].

Table 4 shows standardized *β*-coefficients from the regression analysis of the PCL-5 cluster scores and four dimensions of the mental component of health of the SF-36 questionnaire. The results of univariate regression confirmed that the level of perceived social support was strongly associated with the PTSD symptoms with high statistical significance. A significant association was also observed with all four dimensions of the MHRQoL (*p* < 0.001 for all PCL-5 clusters and MHRQoL indicators). Among all other PCL-5 clusters, perceived social support proved to be the best predictor of the Intrusion (B) subscale of the PTSD symptoms (*β* = −0.478; *p* < 0.001). Of all MHRQoL indicators, the Social functioning dimension was most associated with perceived social support (*β* = −0.437; *p* < 0.001).

Standardized beta coefficients for the Total PCL-5 score and cumulative Mental component score of SF-36 were −0.438 and 0.476, respectively. It confirms that low-perceived social support status was a highly significant predictor of severe PTSD symptoms and poorer QoL.

Similar results have been obtained in numerous other studies. Research dealing with the relationship between social support and concealing emotions from family members indicated the greater likelihood of the development of PTSD in cases where emotions are concealed, the importance of social support from friends, and the inverse relationship between social support and PTSD [45]. Research into HRQoL of Gulf War veterans showed that even 20 years after the war, mental disorders still had a strong effect on QoL, and the influence of social support on QoL was more pronounced in mental than in physical health [41]. A study of the risk factors for PTSD in people exposed to various war traumas alongside other factors (gender, educational, socioeconomic status, etc.) also pointed out the lack of social support at the time of the traumatic event as an important predictor of PTSD [46]. A meta-analysis of 77 studies of risk factors for PTSD indicated the importance of post-traumatic factors, where post-traumatic factors, including social support, were shown to be a more significant predictor than pre-traumatic factors [10].

After including sociodemographic variables (captivity, age, employment, educational, marital, and socioeconomic status) in the regression model, self-perception of the social support remained a highly significant predictor of all PTSD symptoms and MHRQoL (Table 4). However, several other factors also showed a significant association with PCL-5 and SF-36 cluster scores. As expected, age proved to be a significant predictor of the dimensions of Vitality (*β* = −0.169; *p* = 0.005) and Social functioning (*β* = −0.213; *p* < 0.001), indicating a poorer condition for these parameters in older participants.

In addition to self-perceived social support, captivity and socioeconomic status were shown to be strong predictors of PTSD symptoms and MHRQoL. The self-assessed socioeconomic status was associated with C-Avoidance (*β* = −0.166; *p* = 0.025), D-Negative alterations in cognitions and mood (*β* = −0.244; *p* = 0.001), E-Alterations in arousal and reactivity (*β* = −0.279; *p* < 0.001), and Total PCL-5 score (*β* = −0.221; *p* = 0.001). Among all MHRQoL, only the Social functioning was significantly related to the socioeconomic status (*β* = −0.156; *p* = 0.022). These results can be related to the disrupted work functioning of veterans suffering from PTSD and a relatively large number of unemployed veterans and veterans with low retirement income.

The nonimprisoned veteran group was statistically significantly more likely to develop more severe PTSD symptoms compared to ex-POWs, which is evident from the obtained *β*-coefficients for individual clusters: D-Negative alterations in cognitions and mood (*β* = 0.169; *p* = 0.009), and E-Alterations in arousal and reactivity (*β* = 0.236; *p* < 0.001), as well as for the Total PCL-5 score (*β* = 0.169; *p* = 0.008). Similarly, the ex-POW group was found to have better scores for indicators of MHRQoL as well. All dimensions of the MHRQoL were significantly related to captivity status. Standardized beta coefficients for Vitality, Social functioning, Emotional roles limitation, Mental health, and Mental component scores were −0.131, −0.160, −0.133, −0.251, and −0.222, respectively (*p* < 0.05 for all dimensions). As previously assumed in our earlier published paper, the reason why the ex-POWs were in better condition compared to the nonimprisoned veterans regarding PTSD and HRQoL indicators may lie in the fact that more post-war care and social support was provided to ex-POWs through various projects and support programs after their release from captivity. Furthermore, since ex-POWs assessed their socioeconomic status better than nonimprisoned veteran group, their scores for QoL indicators were higher [19].

Numerous studies have addressed various predictors of PTSD and HRQoL of ex-POWs. In a study about ex-POWs of World War II and the Korean War, it was found that the significant predictors of the intensity of PTSD symptoms were age at the time of imprisonment and exposure to combat action, and the most important predictor was shown to be the actual trauma of imprisonment. The next most significant predictor of the severity of PTSD was social support. Here, it was not only pre-war family support, family relationships that were shown to be important, but also close interpersonal relationships in the post-war period. In this study, they concluded that these factors were proven to be important in their effect on the intensity of PTSD, even 45 and 55 years after the war [47]. Research conducted on 270 ex-POWs from the World War II and the Vietnam War, examining various possible predictors of chronic PTSD, showed that the most important predictor of PTSD, even 40 to 50 years after imprisonment, was the severity of the trauma experienced [48], while some studies have indicated the extreme importance of social support [49].

The health of Croatian veterans is still attracting a lot of attention, more than two decades after the war. This population uses the services of the health system to a large extent, and in addition, significant difficulties have been observed in various areas of their functioning. Compared to the general population, the QoL of veterans is reduced. In the context of different variables, studies of the HRQoL in veterans will assist in planning treatment and rehabilitation programs for this population and their families.

### 3.3. Limitations of the Study

Our research has been related to the perception of received social support in the post-war period, but the survey was conducted more than two decades after exposure to trauma. Therefore, in the absence of valid information on the condition of participants until the time when the study was conducted, this research cannot be considered as a true retrospective investigation showing a change in the MHRQoL and intensity of PTSD symptoms over that period of time. Another important limitation to the study was use of a nonstandardized questionnaire about the perceived social support. This questionnaire has been used for years in working with veterans treated at the Psychiatric Department of the County General Hospital Vukovar and Croatian Veterans’ Hospital.

## 4. Conclusions

Self-perceived social support has been identified as an important post-traumatic factor for assessing PTSD symptoms and MHRQoL. The obtained results showed that veterans who had a more positive perception of social support after the events of the war had less intense PTSD symptoms and better MHRQoL. According to the results of regression analysis, the hypothesis of no relationship between perceived social support and PTSD symptoms, as well as MHRQoL, can be rejected.

In addition to self-perceived social support, captivity and socioeconomic status were shown to be strong predictors of PTSD symptoms and MHRQoL. The ex-POW group was more likely to develop less severe PTSD symptoms and have better MHRQoL compared to the nonimprisoned veteran group, probably as a result of more post-war care and social support, which they received after their release from captivity.

## Figures and Tables

**Table 1:** Categorization of self-perceived social support according to the number of positive answers in the questionnaire.

Social Support	Number of Positive (“Yes”) Answers	Subjects *N* (%)
Low	0–1	160 (61.8)
Moderate	2–3	70 (27.0)
High	4–5	29 (11.2)

**Table 2:** Differences in the veterans’ perception of social support associated with different sociodemographic data.

Imprisonment and Sociodemographic Characteristics	Low		Moderate		High				
	(*N*)	(%)	(*N*)	(%)	(*N*)	(%)	*χ* ^2^	*χ^2^_Crit._* (DF) ^1^	*p*
**Imprisoned**									
Yes	44	48.9	32	35.6	14	15.6	9.760	5.991 (2)	0.008 *
No	116	68.6	38	22.5	15	8.9			
**Age**									
<45	17	70.8	7	29.2	0	0.0	17.960	12.592 (6)	0.006 *
45–54	80	62.0	36	27.9	13	10.1			
55–64	59	67.0	17	19.3	12	13.6			
>64	4	22.2	10	55.6	4	22.2			
**Employment status**									
Unemployed	31	79.5	4	10.3	4	10.3	7.616	9.488 (4)	0.107
Employed	24	54.5	14	31.8	6	13.6			
Retired	105	59.7	52	29.5	19	10.8			
**Education**									
Elementary school	40	62.5	12	18.8	12	18.8	19.730	9.488 (4)	0.001 *
High school	111	64.5	44	25.6	17	9.9			
Higher education	9	39.1	14	60.9	0	0.0			
**Marital status**									
Divorced or widower	26	59.1	14	31.8	4	9.1	8.842	9.488 (4)	0.065
Unmarried	29	65.9	15	34.1	0	0.0			
Married	105	61.4	41	24.0	25	14.6			
**Socioeconomic status**									
Bad	63	91.3	6	8.7	0	0.0	114.99	9.488 (4)	<0.001 *
Average	93	57.4	58	35.8	11	6.8			
Good	4	14.3	6	21.4	18	64.3			

^1^ Chi-Square Critical Value (Degrees of Freedom). * *p* < 0.05.

**Table 3:** Differences in PCL-5 cluster scores and SF-36 health survey scores between groups with different perceptions of social support.

Scores		Low		Moderate		High			
		Mean	SD	Mean	SD	Mean	SD	K–W H ^1^	*p*
PCL-5	B	2.6a	0.8	1.8b	0.7	1.4b	0.5	66.042	<0.001 *
	C	2.4a	1.1	2.0b	1.1	1.7b	0.9	17.547	<0.001 *
	D	2.2a	0.9	1.6b	0.8	1.2b	0.6	40.941	<0.001 *
	E	2.2a	0.8	2.0a	0.8	1.2b	0.5	38.331	<0.001 *
	TPCL-5	46.5a	15.3	36.0b	13.7	25.6c	10.6	51.441	<0.001 *
SF-36	VT	27.9b	15.2	32.8ab	18.8	40.5a	17.0	13.532	0.001 *
	SF	31.5c	17.4	40.2b	20.0	61.2a	13.5	51.998	<0.001 *
	RE	5.4b	17.1	26.7a	37.0	32.2a	40.3	39.278	<0.001 *
	MH	34.0b	13.6	40.2a	16.4	46.9a	11.5	22.779	<0.001 *
	MCS	24.7c	10.7	35.0b	17.3	45.2a	11.2	55.005	<0.001 *

B-Intrusion, C-Avoidance, D-Cognition and mood, E-Arousal and reactivity, TPCL-5-Total PCL-5 score, VT-Vitality, SF-Social functioning, RE-Emotional roles limitation, MH-Mental health, MCS Mental component score; K–W H-Kruskal–Wallis H value. The K–W H critical value was 5.992 for all variables. Groups sharing the same letter (a–c) are not significantly different according to Dunn test. * *p* < 0.05.

**Table 4:** Regression analysis and factors associated with the different perception of social support.

Score	Parameter	Univariate		Multivariate							
		SOCSP	*R* ^2^	SOCSP	CAP	AGE	EMP	EDU	MAR	SES	Adj. *R*^2^
B	*β*	−0.478	0.228	−0.453	0.113	0.092	−0.093	−0.026	0.055	−0.031	0.243
	−95% CI	−0.585		−0.582	−0.010	−0.019	−0.212	−0.141	−0.062	−0.165	
	+95% CI	−0.370		−0.324	0.236	0.203	0.026	0.089	0.172	0.102	
	*p*	<0.001 *		<0.001 *	0.071	0.104	0.124	0.654	0.353	0.644	
C	*β*	−0.225	0.050	−0.190	−0.053	0.213	0.083	0.025	0.123	−0.166	0.116
	−95% CI	−0.344		−0.329	−0.185	0.093	−0.045	−0.099	−0.004	−0.310	
	+95% CI	−0.105		−0.050	0.080	0.333	0.212	0.149	0.249	−0.021	
	*p*	<0.001 *		0.008 *	0.434	0.001 *	0.202	0.692	0.057	0.025 *	
D	*β*	−0.393	0.154	−0.255	0.169	0.089	0.091	0.047	0.017	−0.244	0.192
	−95% CI	−0.506		−0.388	0.042	−0.026	−0.032	−0.071	−0.104	−0.382	
	+95% CI	−0.280		−0.122	0.296	0.204	0.214	0.166	0.138	−0.107	
	*p*	<0.001 *		<0.001 *	0.009 *	0.128	0.145	0.434	0.783	0.001 *	
E	*β*	−0.359	0.129	−0.192	0.236	0.032	0.158	0.093	0.160	−0.279	0.201
	−95% CI	−0.473		−0.324	0.110	−0.082	0.036	−0.024	0.040	−0.416	
	+95% CI	−0.244		−0.059	0.363	0.146	0.279	0.211	0.280	−0.142	
	*p*	<0.001 *		0.005 *	<0.001 *	0.585	0.012 *	0.120	0.009 *	<0.001 *	
TPCL-5	*β*	−0.438	0.192	−0.316	0.169	0.101	0.073	0.045	0.090	−0.221	0.223
	−95% CI	−0.548		−0.447	0.045	−0.012	−0.047	−0.071	−0.029	−0.356	
	+95% CI	−0.327		−0.185	0.293	0.213	0.194	0.162	0.208	−0.085	
	*p*	<0.001 *		<0.001 *	0.008 *	0.079	0.230	0.442	0.137	0.001 *	
VT	*β*	0.227	0.051	0.194	−0.131	−0.169	−0.197	0.067	0.271	0.080	0.159
	−95% CI	0.107		0.058	−0.261	−0.286	−0.322	−0.054	0.148	−0.060	
	+95% CI	0.346		0.330	−0.002	−0.052	−0.072	0.188	0.395	0.221	
	*p*	<0.001 *		0.005 *	0.047 *	0.005 *	0.002 *	0.278	<0.001 *	0.262	
SF	*β*	0.437	0.191	0.367	−0.160	−0.213	−0.105	−0.022	0.042	0.156	0.240
	−95% CI	0.326		0.237	−0.283	−0.325	−0.224	−0.137	−0.075	0.022	
	+95% CI	0.547		0.496	−0.037	−0.102	0.014	0.093	0.160	0.290	
	*p*	<0.001 *		<0.001 *	0.011 *	<0.001 *	0.084	0.711	0.476	0.022 *	
RE	*β*	0.376	0.141	0.301	−0.132	0.045	0.034	−0.101	−0.110	0.085	0.153
	−95% CI	0.262		0.164	−0.262	−0.072	−0.091	−0.222	−0.234	−0.056	
	+95% CI	0.490		0.437	−0.002	0.163	0.160	0.021	0.014	0.227	
	*p*	<0.001 *		<0.001 *	0.046 *	0.448	0.592	0.104	0.081	0.235	
MH	*β*	0.292	0.085	0.241	−0.251	−0.048	−0.037	0.131	0.279	0.022	0.232
	−95% CI	0.175		0.111	−0.375	−0.159	−0.157	0.016	0.161	−0.113	
	+95% CI	0.410		0.371	−0.128	0.064	0.082	0.247	0.397	0.156	
	*p*	<0.001 *		<0.001 *	<0.001 *	0.403	0.538	0.026 *	<0.001 *	0.750	
MCS	*β*	0.476	0.227	0.392	−0.222	−0.111	−0.085	−0.005	0.109	0.125	0.285
	−95% CI	0.368		0.267	−0.341	−0.219	−0.200	−0.117	−0.005	−0.005	
	+95% CI	0.584		0.518	−0.103	−0.003	0.030	0.107	0.222	0.254	
	*p*	<0.001 *		<0.001 *	<0.001 *	0.044 *	0.148	0.931	0.061	0.060	

*β*-standardized beta coefficient, CI-Confidence interval, PCL-5 clusters (B-Intrusion, C-Avoidance, D-Cognition and mood, E-Arousal and reactivity, TPCL-5-Total PCL-5 score), SF-36 dimensions (VT-Vitality, SF-Social functioning, RE-Emotional roles limitation, MH-Mental health, MCS-Mental component score), SOCSP-Social support, CAP-Captivity, AGE-Age of participants; EMP-Employment, EDU-Education, MAR-Marital status, SES-Socioeconomic status; * *p* < 0.05; Adj. *R*^2^-Adjusted *R*^2^.

## Supplementary Materials

The following are available online at https://www.mdpi.com/2227-9032/8/4/396/s1, Figure S1: The perceived social support questionnaire.
