# Supplementary material for: The Role of Perceived Social Support in Assessing Posttraumatic Stress Disorder and Mental Health-Related Quality of Life in Veterans
Source: Healthcare (Basel). 2020 Oct 12;8(4):396. doi: 10.3390/healthcare8040396 (PMC7711990; doi:10.3390/healthcare8040396)
Supplement: Supplementary file 1 [file healthcare-08-00396-s001.pdf]

Patient’s code:_________________ Date:__________________

SOCIAL SUPPORT QUESTIONNAIRE

The questions relate to your experience of the support you had or felt upon your return from the battlefield and/or from captivity.

Please answer the following questions by marking one answer for each question.

1. Did you have support and understanding from your family (wife, children, parents, siblings or other family members) regarding your problems and needs?

   ☐ Yes ☐ No

2. Did you have support and understanding from your close friends regarding your problems and needs?

   ☐ Yes ☐ No

3. Did you have support and understanding from other important people (priest, work colleagues, neighbours or others) in life regarding your problems and needs?

   ☐ Yes ☐ No

4. Did you have support and understanding from your comrades or veterans organizations regarding your problems and needs?

   ☐ Yes ☐ No

5. Did you have support and understanding from social community (local institutions, health institutions, state institutions and others from whom you have requested assistance) regarding your problems and needs?

   ☐ Yes ☐ No
